# STING deficiency promotes motor recovery in mice following brachial plexus root avulsion

**DOI:** 10.1002/ame2.70114

**Published:** 2025-12-09

**Authors:** Yu Peng, Ying Zhang, Shenhui Yang, Lu He, Shuangxi Chen

**Affiliations:** ^1^ Department of Neurology, Multi‐Omics Research Center for Brain Disorders, The First Affiliated Hospital, Hengyang Medical School University of South China Hengyang Hunan China; ^2^ Clinical Research Center for Immune‐Related Encephalopathy of Hunan Province (The First Affiliated Hospital), Hengyang Medical School University of South China Hengyang Hunan China; ^3^ Institute of Pharmacy and Pharmacology, Hunan Provincial Key Laboratory of Tumor Microenvironment Responsive Drug Research, Hunan Province Cooperative Innovation Center for Molecular Target New Drug Study University of South China Hengyang Hunan China; ^4^ Department of Neurosurgery, The First Affiliated Hospital, Hengyang Medical School University of South China Hengyang Hunan China

**Keywords:** brachial plexus root avulsion (BPRA), motoneuron (MN), neuroinflammation, synthase‐stimulator of interferon genes (STING)

## Abstract

**Background:**

Brachial plexus root avulsion (BPRA), a well‐known form of peripheral nerve injury, results in motor function loss in the affected forelimb due to motoneuron (MN) death, which may be influenced by neuroinflammation following a lesion in the spinal cord. Although synthase‐stimulator of interferon genes (STING) signaling can contribute to chronic inflammation and tissue damage in a number of pathological conditions, the essential role of STING signaling in BPRA remains to be reported. Based on our previous findings that the STING mRNA level is upregulated in the anterior horn of the segment of the affected spinal cords of mice with BPRA, STING may be associated with motor recovery in BPRA.

**Methods:**

In the present study, STING knockout transgenic mice were used to establish a BPRA re‐implantation model, which was followed by behavioral tests, histochemical staining and quantitative reverse transcription polymerase chain reaction.

**Results:**

The results demonstrated that STING deficiency can increase the body weight, promote motor recovery, decrease MN death, inhibit pyroptosis and neuroinflammation, increase remyelination, and reduce the atrophy of the biceps brachii in mice with BPRA.

**Conclusion:**

These combined results suggest that inhibition of STING may be a promising strategy for treating BPRA.

## Graphical abstract

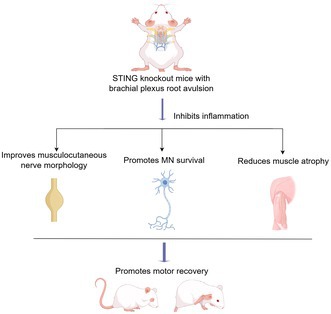

## 1 INTRODUCTION

Brachial plexus injury (BPI), a widely‐known form of peripheral nerve injury that can be caused by birth injury, cervical spine procedures or traffic accidents, can result in serious dysfunction and significant morbidity in the population.^1^ In cases of BPI, approximately 21% of individuals suffer from brachial plexus root avulsion (BPRA) and most patients suffer from multiple nerve root avulsions, leading to the permanent loss of motor function,^2^, ^3^ which is considered one of the most severe types of upper limb nerve injury.^4^ Although re‐implantation surgery is widely performed in the clinic to treat this injury, motor function cannot be entirely restored due to the very slow axonal re‐growth of motoneurons (MNs) in the anterior horn of the affected spinal cord, which is required to re‐innervate the biceps brachii before the atrophy of target muscles.^5^, ^6^ Hence, MN survival is essential for motor recovery in the corresponding limb, and early strategies that focus on promoting the survival of lesioned MNs are urgently needed.^7^, ^8^ Additionally, the early pathological changes associated with the mechanism of BPRA involve inflammation and apoptosis, which cause notable neuronal death.^9^, ^10^ Therefore, effective anti‐inflammatory strategies that can reduce neuronal death may treat BPRA.

Attempts have been made to elucidate the target that could inhibit neuroinflammation to prevent MN death. One potential candidate is stimulator of interferon genes (STING; TMEM173), an endoplasmic reticulum‐localized adaptor and essential molecule that can trigger the immune response.^11^ Induction of STING can result in an inflammatory cascade that releases multiple proinflammatory mediators via activating the NF‐κB and type I interferon (IFN) pathways,^12^ leading to a cycle of inflammation and tissue damage, and consequently exacerbating the progression and severity of disorders.^13^, ^14^ Besides its main functions in microbial pathogens, STING can also induce IFN signaling in neuronal immunomodulation.^15^, ^16^ Furthermore, blocking the STING signaling pathway can inhibit apoptosis, reduce inflammation and oxidative stress, and slow down pathological damage in lung ischemia/reperfusion rats.^17^

In the present study, it was demonstrated for the first time that genes controlled by the STING pathway are upregulated in the ventral horn following BPRA. Genetic ablation of STING promoted motor recovery, decreased MN death, increased remyelination, and reduced muscle atrophy in mice subjected to BPRA. These results provide important insights into the role of STING after BPRA and may inform novel therapeutic strategies for treating this type of injury.

## 2 MATERIALS AND METHODS

### 2.1 Animals

MPYS/STING knockout (KO) mice (Sting, stock no. 025805; Jackson Laboratory, USA) harboring a targeted deletion of exons 3–5 in the *Tmem173* gene were purchased. Mice were maintained under a controlled 12‐h light/12‐h dark cycle at 21℃ ± 2℃. Water and food were provided ad libitum. All animal experiments received ethical approval from The Laboratory Animal Ethics Committee of The First Affiliated Hospital of University of South China (permit no. USC2024XS286).

### 2.2 BPRA and re‐implantation model

**FIGURE 1:**
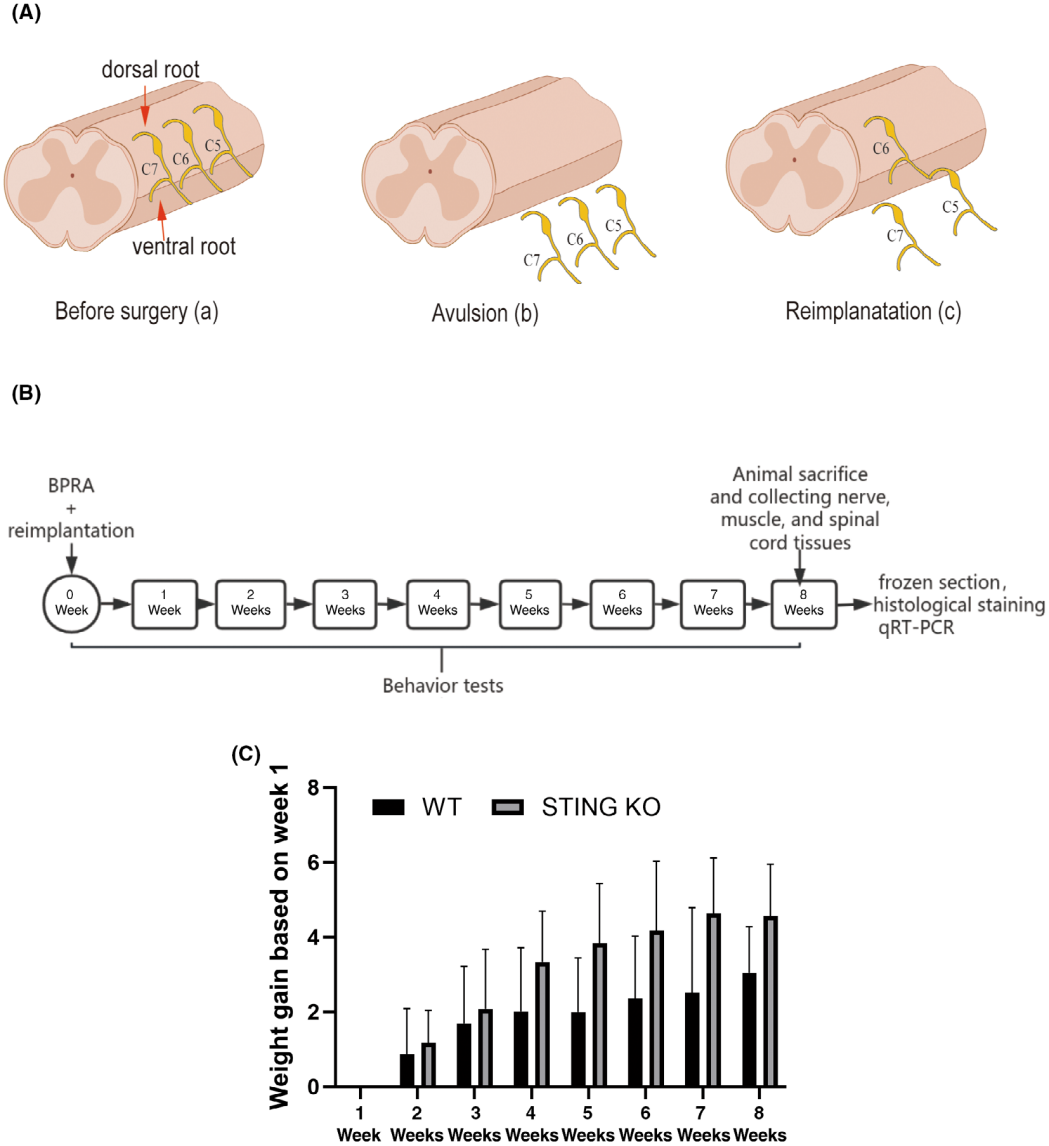
BPRA and reimplantation mouse model, experimental design and body weight after BPRA in mice. (A) Schematic drawing of the procedures for BPRA and reimplantation mouse model. (a) Normal structure of C5–C7 segments in spinal cord before surgery; (b) the avulsion of C5–C7 ventral root and dorsal root; (c) the reimplantation of the C6 ventral root into the corresponding spinal cord segment. (B) The workflow of the experimental procedure. (C) The average body weight gain over weeks 1–8, based on week 1 is shown (*n* = 7).

The animal procedures followed previous studies with slight adjustments^18^, ^19^, ^20^ (Figure 1A). Adult male C57BL/6 wild‐type (WT) and STING^−/−^ mice (8 weeks old; 15 ± 3 g) were used to establish the BPRA model. Mice were anesthetized via intraperitoneal administration of avertin (cat. no. JT0781; JITIAN BIO, China) at a dose of 13 μL/g and then placed in a prone position on an operating platform. Upon withdrawal of the paravertebral muscles, the C5–C7 segments in the spinal cord were localized using the T2 spinous process as an anatomical landmark. A right‐sided hemilaminectomy spanning the C4–C6 vertebrae was performed, after which the dura mater was incised and the right dorsal and ventral roots in the C5–C7 segments were avulsed. To prevent reinnervation, the proximal 2‐mm segments of these nerve roots were surgically excised. In the re‐implantation models, the C6 ventral root was promptly reattached to its original avulsion site on the pial surface. At 24 h post‐surgery, all mice exhibited a Terzis grooming test (TGT) score of 0, confirming successful surgery and the loss of motor function. Animals were allocated into two groups (*n* = 12 per group): a WT group and a STING KO group.

### 2.3 Behavioral tests

Behavioral assessments, including the TGT and cylinder test, were conducted weekly (Figure 1B).

### 2.4 TGT

**FIGURE 2:**
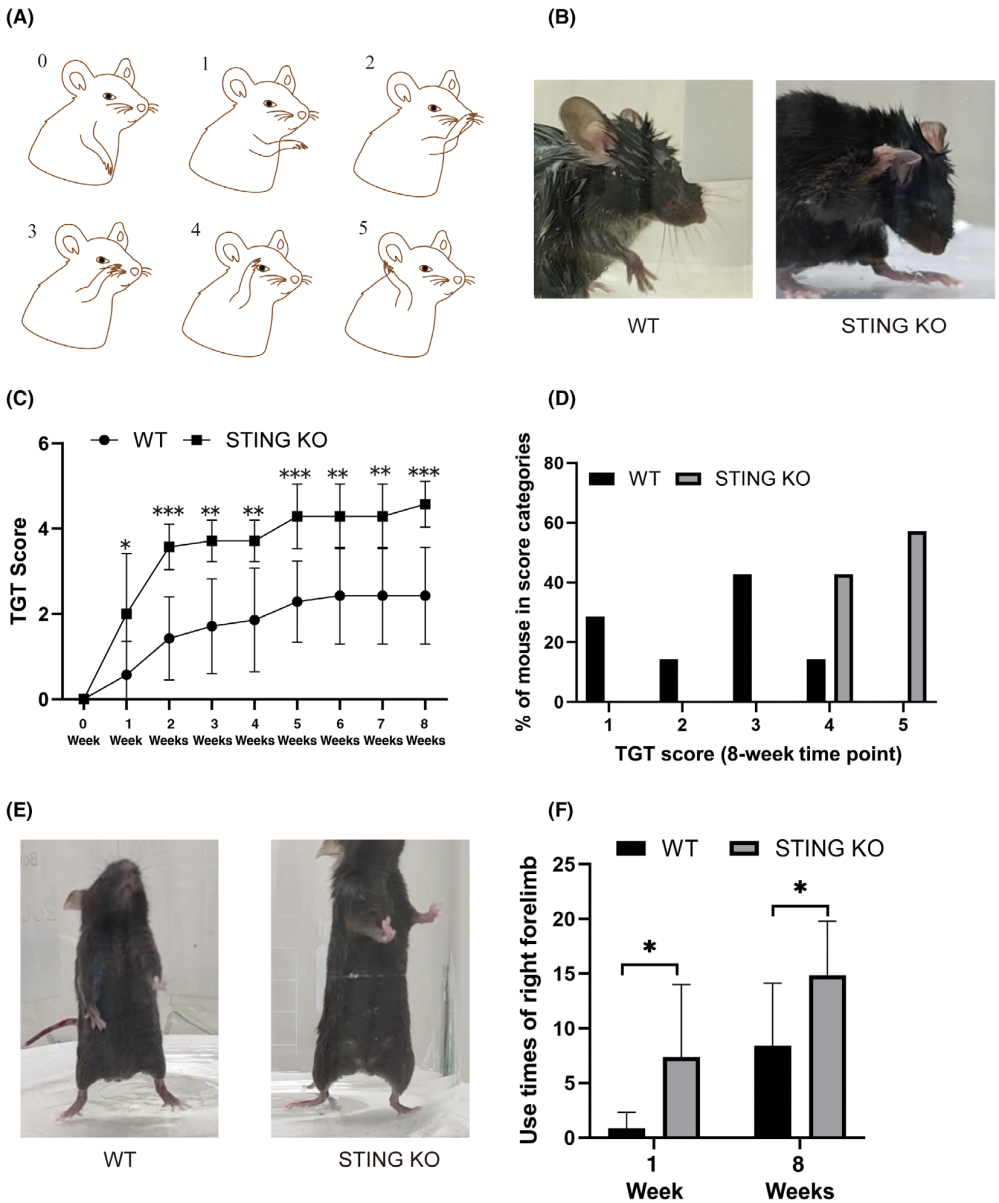
Effect of STING deficiency on motor recovery of the injured forelimb after BPRA in mice. (A) The rating criterion for the TGT. (B) Representative images of TGT in mice at the 8‐week time point postoperatively. (C) Average TGT scores were increased in response to the STING deficiency after BPRA at each time point (*n* = 5, ***p < 0.001, **p < 0.01, *p < 0.05). (D) The proportion of TGT score category at 8 weeks postoperatively. (E) Representative images of the cylinder test in mice at the 8‐week time point postoperatively. (F) Averaged use times of right forelimb at 1‐week and 8 weeks time points postoperatively (*n* = 7, *p < 0.05).

Following surgery, the TGT (*n* = 7 per group) was conducted weekly using a double‐blind method to assess motor function recovery, following established protocols.^21^, ^22^ The procedure involved spraying sterile deionized water onto the heads of the mice, followed by placing each mouse individually in a cylindrical glass container. The mice were then observed for 5 min and the forepaw contact with the head as mice naturally flexed their limbs to remove the water droplets was recorded. The highest forelimb movement score within the 5‐min period was recorded using pre‐established criteria listed in Table 1 and Figure 2A.

**TABLE 1:** TGT test scoring criteria.

Upper Limb Position	Score
No movement	0 point
Elbow flexion reflex present but unable to touch the nose	1 point
Elbow flexion reflex present and able to touch the nose	2 points
Elbow flexion reflex present and able to touch below the eye	3 points
Elbow flexion reflex present and able to touch the eye	4 points
Elbow flexion reflex present and able to touch the ear or behind the ear	5 points

### 2.5 Cylinder test

The cylinder test (*n* = 7 per group) was conducted to assess the recovery of forelimb neuromuscular coordination function as previously detailed.^23^ Mice were individually placed in a 50 cm‐diameter, 70 cm‐high glass cylinder. Then, high‐definition video recordings (30 fps) were used to quantify the right forepaw contacts with the cylinder wall until 20 left forepaw contacts occurred.

### 2.6 Quantitative reverse transcription polymerase chain reaction (qRT‐PCR)

Eight weeks post‐surgery, the mice were euthanized by isoflurane (YUYAN, China). Then, the right side ventral horn of the C5‐C7 segments of the spinal cord tissues were collected (Figure 1B) and qRT‐PCR was performed as previously described.^24^, ^25^ Total RNA was extracted from the spinal cord tissues by RNAiso Plus (cat. no. G3013; Wuhan Servicebio Technology Co., Ltd., China). Reverse transcription was performed on all samples using Hifair III 1st Strand cDNA Synthesis SuperMix (cat. no. 11141ES10; Shanghai Yeasen Biotechnology Co., Ltd., China). qPCR analyses were conducted using SYBR Green‐based (cat. no. 11201ES03; Shanghai Yeasen Biotechnology Co., Ltd., China) detection, adhering strictly to the manufacturer's recommended experimental protocols. The primer sequences are listed in Table 2.

**TABLE 2:** Primer sequences for qRT‐PCR.

Genes	Towards	Sequences (5′‐3′)
ngfr	F	CTAGGGGTGTCCTTTGGAGGT
	R	CAGGGTTCACACACGGTCT
sox2	F	GCGGAGTGGAAACTTTTGTCC
	R	CGGGAAGCGTGTACTTATCCTT
Sting	F	GGTCACCGCTCCAAATATGTAG
	R	CAGTAGTCCAAGTTCGTGCGA
GFAP	F	CGGAGACGCATCACCTCTG
	R	AGGGAGTGGAGGAGTCATTCG
IL‐1β	F	GCAACTGTTCCTGAACTCAACT
	R	ATCTTTTGGGGTCCGTCAACT
IL‐6	F	CCAAGAGGTGAGTGCTTCCC
	R	CTGTTGTTCAGACTCTCTCCCT
TNF‐α	F	CCCTCACACTCAGATCATCTTCT
	R	GCTACGACGTGGGCTACAG
NLRP3	F	ATTACCCGCCCGAGAAAGG
	R	TCGCAGCAAAGATCCACACAG
IL‐18	F	GACTCTTGCGTCAACTTCAAGG
	R	CAGGCTGTCTTTTGTCAACGA
Fas	F	TATCAAGGAGGCCCATTTTGC
	R	TGTTTCCACTTCTAAACCATGCT
Caspase‐3	F	TGGTGATGAAGGGGTCATTTATG
	R	TTCGGCTTTCCAGTCAGACTC
bax	F	TGAAGACAGGGGCCTTTTTG
	R	AATTCGCCGGAGACACTCG
Caspase‐1	F	ACAAGGCACGGGACCTATG
	R	TCCCAGTCAGTCCTGGAAATG
Caspase‐4	F	ACAAACACCCTGACAAACCAC
	R	CACTGCGTTCAGCATTGTTAAA
GSDMD	F	CCATCGGCCTTTGAGAAAGTG
	R	ACACATGAATAACGGGGTTTCC
18S	F	AAACGGCTACCACATCCAAG
	R	TACAGGGCCTCGAAAGAGTC

### 2.7 Histochemical staining

Eight weeks post‐surgery, the mice were euthanized by isoflurane as described above, the thoracic cavity was opened to expose the heart and a needle was introduced into the left ventricle. The heart was perfused with PBS (cat. no. G0002; Wuhan Servicebio Technology Co., Ltd., China) to remove the blood, followed by 4% PFA (cat. no. G1101; Wuhan Servicebio Technology Co., Ltd., China) for fixation. The C5‐C7 segments of the spinal cord tissues, along with the musculocutaneous nerves and biceps brachii muscles, were surgically isolated (Figure 1B). The muscle mass was quantified using a precision balance. All tissues were fixed in 4% PFA overnight. The tissues then underwent graded sucrose dehydration (15%, 30% and 40% solutions) (cat. no. GC205014; Wuhan Servicebio Technology Co., Ltd., China) where they were transferred to each solution until they sank. The dehydrated tissues were embedded in OCT compound (cat. no. G6059; Wuhan Servicebio Technology Co., Ltd., China) and stored at −80℃. Sections of the tissues were collected using a Leica CM 1950 microtome.

The survival rate of the spinal MNs was quantified through Nissl staining using 1% neutral red (cat. no. G1318; Beijing Solarbio Science & Technology Co., Ltd., China). Frozen C5–C7 spinal cord sections were cut to a thickness of 10 μm and then stained with 1% neutral red solution for 1 h. Subsequently, the sections underwent sequential dehydration through an ethanol (cat. no. A500737; Sangon Biotech Co., Ltd., China) series and xylene (cat. no. A530011; Sangon Biotech Co., Ltd., China). The sections were then mounted with neutral resin (cat. no. WG10004160; Wuhan Servicebio Technology Co., Ltd., China) and polymerized at 37℃ overnight.

For Luxol fast blue (LFB) staining, transverse musculocutaneous nerve sections (5 μm) were incubated in LFB solution (cat. no. DK0008; Leagene; Beijing Regen Biotechnology Co., Ltd., China) at 37℃ for 24 h. Then the sections were washed successively in 95% ethanol, 0.1% lithium carbonate solution (cat. no. DK0008; Leagene; Beijing Regen Biotechnology Co., Ltd., China), 70% ethanol, and water. Subsequently, the slides were dehydrated through a graded series of ethanol solutions (95% and 100%), were cleared in xylene and were finally covered with a coverslip using neutral balsam.

For immunofluorescence staining, 5‐μm musculocutaneous nerve sections were washed three times with PBS containing 0.1% Triton X‐100 (cat. no. 20107ES76; Shanghai Yeasen Biotechnology Co., Ltd., China) and then incubated with anti‐choline acetyltransferase (ChAT; 1:50; cat. no. ab181023; Abcam, UK) antibody at 4℃ for 16–18 h. Subsequently, the sections were incubated with an Alexa Fluor 488‐conjugated secondary antibody (1:100; cat. no. ab150077; Abcam, UK) for 60 min at 25℃. Then the sections were washed and mounted with solution containing 4′,6‐diamidino‐2‐phenylindole (cat. no. P36935; Gibco; Thermo Fisher Scientific, Inc., USA).

For hematoxylin and eosin (H&E) staining, the biceps brachii sections were incubated in hematoxylin (cat. no. G1120‐100; Beijing Solarbio Science & Technology Co., Ltd., China) for 2 min to stain the nuclei, followed by differentiation in 0.5% hydrochloric acid‐ethanol to refine the nuclear staining. Subsequently, the sections were stained with eosin (cat. no. G1120‐100; Beijing Solarbio Science & Technology Co., Ltd., China) for 5 s to visualize the cytoplasm. Finally, the sections were dehydrated thrice in absolute ethanol, for 5 min each.

Histological analysis of the Nissl, LFB and H&E‐stained specimens (all *n* = 3) was conducted using a Leica microscope, with the MN populations and fibroblast nuclei systematically quantified using ImageJ 5.0 (National Institutes of Health) following digital image calibration. The immunofluorescence‐stained sections were imaged under an AxioObserver A1 microscope (Carl Zeiss AG, Germany) using AxioVision 4.6 software (Carl Zeiss AG, Germany).

### 2.8 Statistical analysis

The results are presented as the mean ± standard deviation. Statistical evaluations were executed utilizing GraphPad Prism 9.0 software (Dotmatics). Data were analyzed using the Student's *t* test. Statistical significance was set at *p* < 0.05.

## 3 RESULTS

### 3.1 The STING mRNA levels were upregulated in the right side anterior horn of the C5‐C7 spinal cord segments from the BPRA mouse model

**FIGURE 3:**
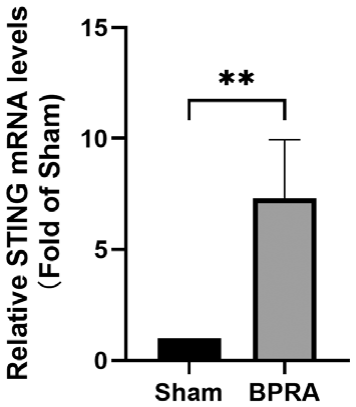
Change of STING at the mRNA level in the ventral horn after BPRA in mice. STING mRNA level was up‐regulated after BPRA after injury for 8 weeks (*n* = 4, **p < 0.01).

qRT‐PCR was conducted to evaluate the role of STING in mice following BPRA. Compared with the Sham group, the STING mRNA levels were significantly upregulated after BPRA (Figure 3).

### 3.2 STING deficiency increased the body weight of the BPRA mouse model

To verify the impact of STING deficiency on the somatic growth patterns of mice with BPRA, body weight measurements were collected weekly following lesion. Compared with the WT group, the body weight of the STING KO group showed an increase (Figure 1C).

### 3.3 STING deficiency promoted motor recovery in the BPRA mouse model

To investigate whether STING deficiency can improve motor recovery in mice with BPRA, TGTs and cylinder tests were performed weekly. Compared with the WT group, the mean TGT scores in the STING KO group exhibited a marked upward trend from the 1st to the 8th week (Figure 2B,C). Additionally, at 8 weeks post injury, compared with the WT group, the proportion of scores at 4 and 5 was markedly elevated in the STING KO group (Figure 2D). In the cylinder test, compared with the WT group, the right forelimb use times were significantly increased in the STING KO group at the 1st and 8th weeks (Figure 2E,F).

### 3.4 STING deficiency reduced MN death in the BPRA mouse model

**FIGURE 4:**
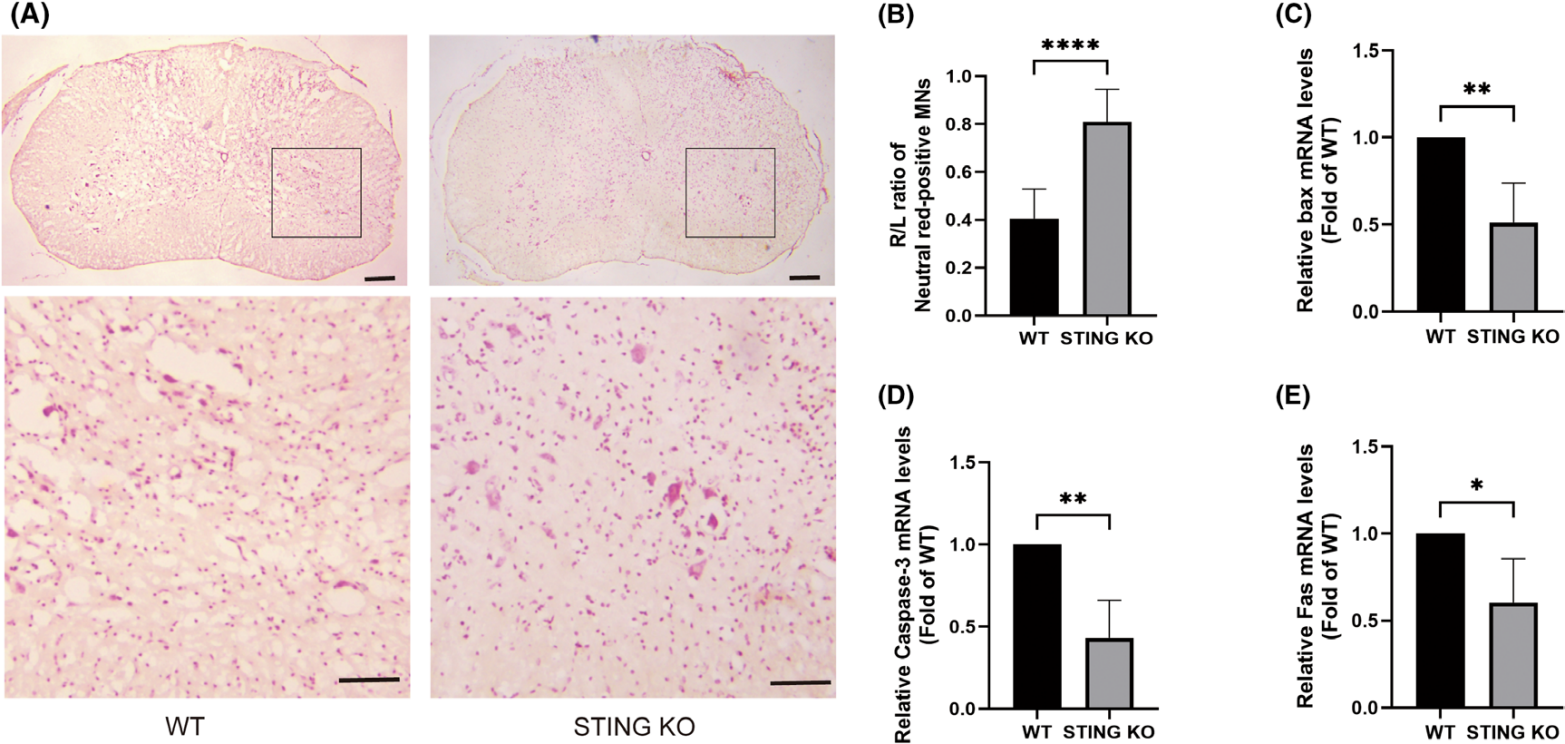
Effect of STING deficiency on MN survival in spinal cord after BPRA in mice. (A) Representative images of C5–C7 segments of spinal cord sections stained with neutral red. Scale bars represent 20 μm. (B) The MN survival rate was calculated as the injury (R)/intact (L) ratio of MNs located in the ventral horn. (C–E) The mRNA levels of bax (C), Caspase‐3 (D) and Fas (E) in the ventral horn were decreased in response to the STING deficiency after BPRA (*n* = 3, ****p < 0.0001, **p < 0.01, *p < 0.05).

To evaluate the influence of STING deficiency on MN viability in mice with BPRA, neutral red staining and qRT‐PCR were used to quantify the MN survival rate and the expression of apoptosis‐related genes in the lesioned ventral horn. Compared with the WT group, the number of MNs (Figure 4A,B) were increased in the STING KO group. Additionally, compared with the WT group, the mRNA levels of Bax (Figure 4C), Caspase‐3 (Figure 4D) and Fas (Figure 4E) were decreased in the STING KO group.

### 3.5 STING deficiency inhibited pyroptosis and neuroinflammation in the BPRA mouse model

**FIGURE 5:**
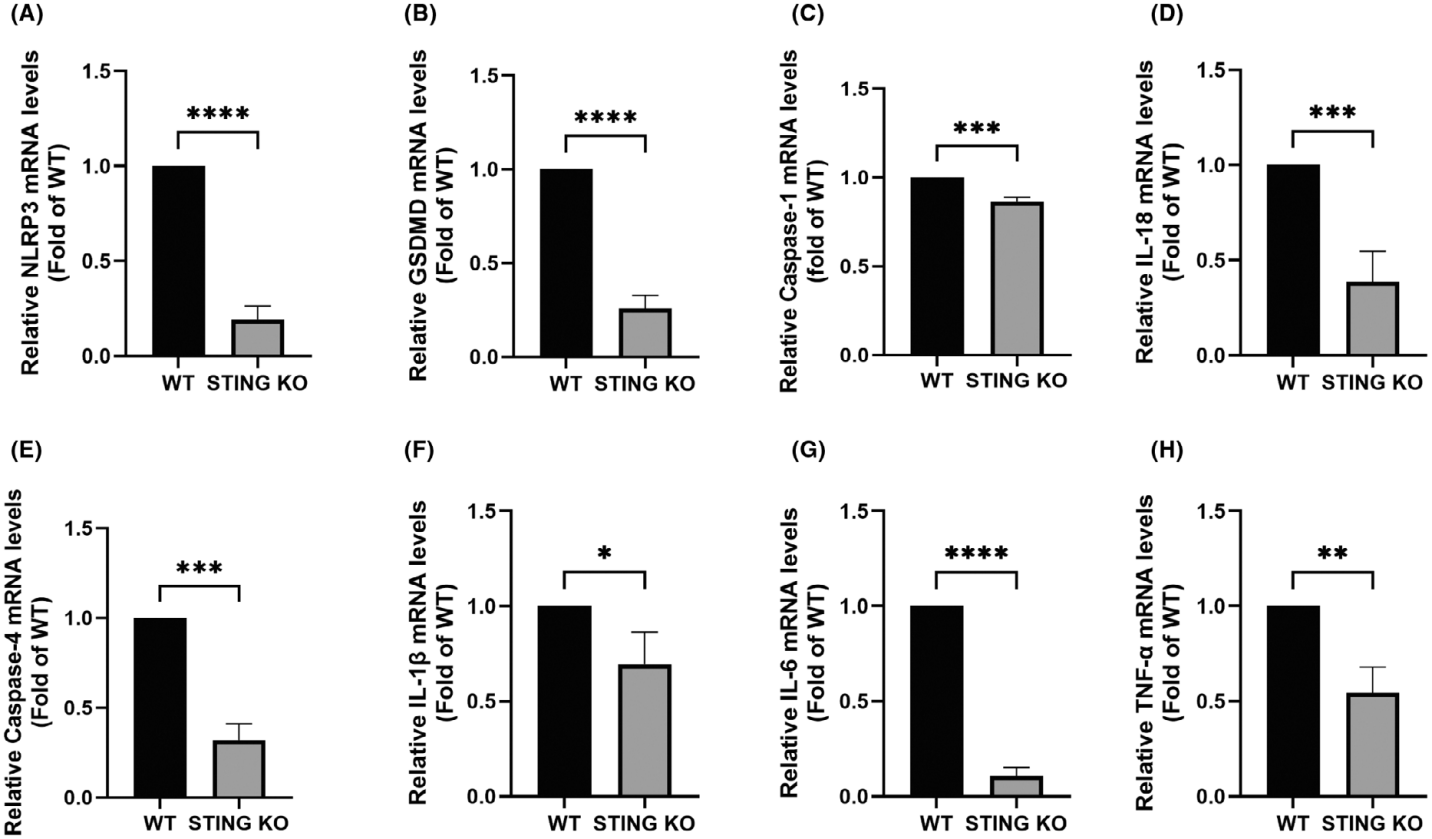
Effect of STING deficiency on the inflammation and pyroptosis in ventral horn of spinal cord after BPRA in mice. The mRNA levels of NLRP3 (A), GSDMD (B), caspase‐1 (C), IL‐18 (D), Caspase‐4 (E), IL‐18 (F), IL‐6 (G), and TNF‐α (H) in the ventral horn were decreased in response to the STING deficiency after BPRA (*n* = 3, ****p < 0.0001, ***p < 0.001, **p < 0.01, *p < 0.05).

To elucidate the mechanistic basis underlying the neuroprotective effect of STING deficiency on MN survival in mice with BPRA, qRT‐PCR was used to quantify the relative mRNA expression levels of pyroptosis and inflammation‐related genes in the anterior horn of the injured spinal cord. Compared with the WT group, the mRNA levels of NLR family pyrin domain containing 3 (NLRP3) (Figure 5A), Gasdermin D (Figure 5B), Caspase‐1 (Figure 5C), IL‐18 (Figure 5D), Caspase‐4 (Figure 5E), IL‐1β (Figure 5F), IL‐18 (Figure 5G) and TNF‐α (Figure 5H) were decreased in the STING KO group.

### 3.6 STING deficiency increased the number of MN axons and remyelination in the musculocutaneous nerve of the BPRA mouse model

**FIGURE 6:**
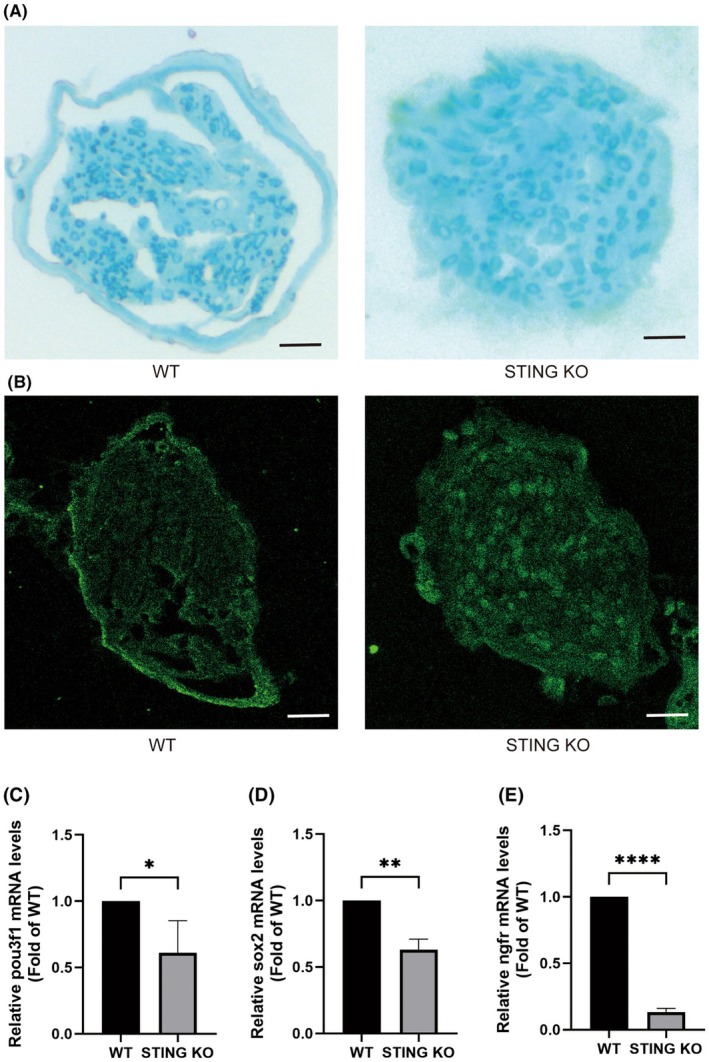
Effect of STING deficiency on histological alterations and the remyelination in musculocutaneous nerves after BPRA in mice. (A) Histological images of musculocutaneous nerve sections with LFB staining. Scale bars represent 20 μm. (B) Histological images of musculocutaneous nerve sections with immunofluorescence staining of ChAT. (C–E) The mRNA levels of pou3f1 (C), sox2 (D) and ngfr (E) were in the ventral horn were decreased in response to the STING deficiency after BPRA (*n* = 3, ****p < 0.0001, **p < 0.01, *p < 0.05).

To systematically evaluate the impact of STING deficiency on the morphology of the myocutaneous nerve and remyelination in mice with BPRA, LFB and ChAT immunofluorescence staining of myocutaneous nerves and qRT‐PCR analysis of demyelination‐related genes in the anterior horn of the injured spinal cord were performed. Compared with the WT group, the number of LFB‐positive and ChAT‐positive axons were increased in the STING KO group (Figure 6A,B). Additionally, compared with the WT group, mRNA levels of POU4F1 (Figure 6C), sox2 (Figure 6D) and NGFR (Figure 6E) were decreased in the STING KO group.

### 3.7 STING deficiency prevented muscle atrophy in the BPRA mouse model

**FIGURE 7:**
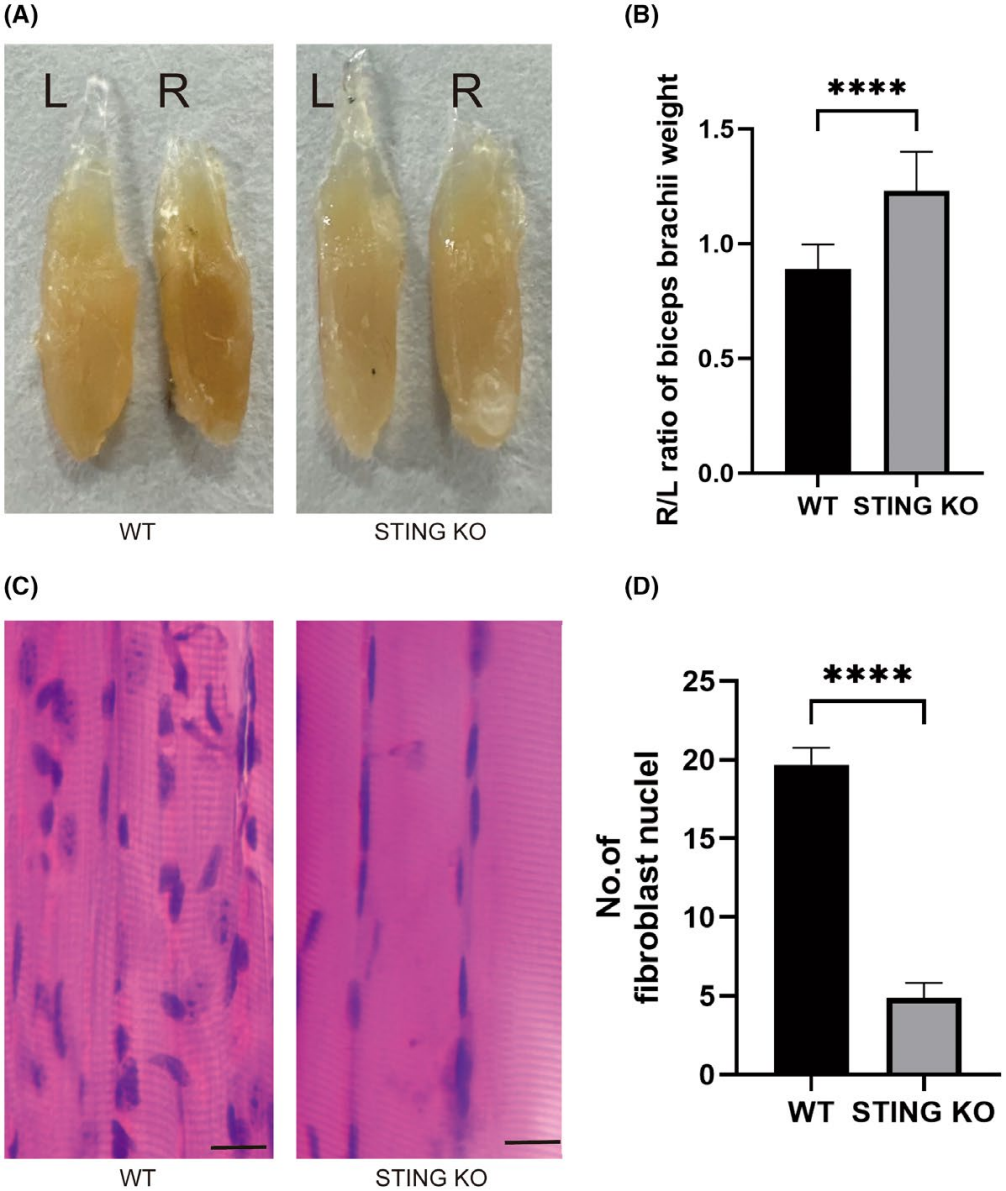
Effect of STING deficiency on the atrophy in biceps after BPRA in mice. (A) Representative images of biceps muscles. (B) The muscle atrophy was calculated as the injury (R)/intact (L) ratio of muscle weight at 8 weeks postoperatively. (C) Representative images of longitudinal bicep muscle sections with H&E staining. Scale bars represent 100 μm. (D) The extent of fibrosis was calculated as the injury (R)/intact (L) ratio of the number of fibroblast nuclei at 8 weeks postoperatively (*n* = 3, ****p < 0.0001).

To determine the modulatory effect of STING deficiency on muscle atrophy in mice with BPRA, the biceps brachii were weighed, H&E staining was performed and the number of fibroblast nuclei were calculated. Compared with the WT group, the weight of the biceps brachii muscles was increased (Figure 7A,B) and the number of fibroblast nuclei was decreased (Figure 7C,D) in the STING KO group.

## 4 DISCUSSION

Although neurosurgical repair strategies are widely performed to restore anatomical continuity, motor recovery after BPRA is often poor.^26^, ^27^ Hence, combining multiple treatments is necessary for promoting motor function recovery after nerve replantation following BPRA.^28^ In our previous studies, we reported that edaravone,^20^ acetylglutamine,^23^ artemisinin,^22^ berberine^29^ and neuregulin‐1^18^ can accelerate motor function recovery in mouse or rat models following BPRA. The results of the present study indicated that STING deficiency can suppress neuroinflammatory responses and MN death to facilitate motor recovery in a mouse model of BPRA.

BPI can result in the loss of arm function, which severely affects the quality of life of patients. Hence, motor function recovery is an essential indicator and the main goal of therapeutic evaluation for injury. The present study identified that STING deficiency can facilitate motor function recovery.

When avulsion occurs, severe progressive MN death in the spinal cord is a notable impediment for motor recovery,^30^, ^31^ and lesioned MNs are compelled to initiate survival mechanisms and undergo axonal regeneration.^5^, ^31^ Maintaining MN survival following lesion is necessary for functional restoration. In the present study, it was demonstrated that STING deficiency can reduce the death of MNs. Following root avulsion, a complex pathophysiological cascade, including apoptosis and inflammation, results in the significant degeneration of MNs in the affected segments of spinal cords.^32^ Through qRT‐PCR analysis, the present study demonstrated that STING deficiency can downregulate the mRNA levels of Bax, Caspase‐3 and Fas.

Following spinal root avulsion, proinflammatory cytokines are generated by activating astrocytes and microglia^33^, ^34^ to inhibit the survival of neurons.^35^ Pyroptosis, a widely‐known type of regulated cell death, is closely linked to inflammatory cytokine production. Additionally, the cGAS‐STING signaling pathway can activate the NLRP3 and downstream inflammatory signaling pathways.^36^, ^37^ In the present study, it was revealed that STING deficiency can downregulate the mRNA levels of inflammation and pyroptosis markers.

The motor innervation of both biceps brachii heads is exclusively provided by the musculocutaneous nerve (C5–C7 roots). MN axons release acetylcholine from their terminal boutons within the neuromuscular junction, thereby mediating trophic signaling, which regulates the survival and synaptic maintenance of associated muscle fibers. In the rat BPRA model, regenerated axons undergo efficient remyelination, which researchers have linked to functional recovery during the acute phase.^38^ Experimental evidence from the present study demonstrates that STING deficiency can promote remyelination.

The biceps muscle is mainly influenced by the lateral bundle of the brachial plexus.^39^ Following the degeneration of MNs and the disruption of neuromuscular junction targeting, impaired innervation muscle fibers undergo compensatory reinnervation.^40^ Long‐term denervation results in muscle atrophy, characterized by a reduction in muscle weight and increased levels of fibroblasts.^39^, ^41^ In the present study, it was revealed that STING deficiency can reduce muscle atrophy.

In summary, the results of the present study indicated that STING deficiency may inhibit inflammation to reduce MN death, enhance axonal remyelination and mitigate muscle atrophy in the biceps brachii to improve motor restoration in the BPRA mouse model. However, the present study has several limitations due to the difficulty in breeding STING KO mice. Further studies are therefore needed to elucidate the potential molecular mechanisms by which STING deletion may contribute to motor neuron protection, to position STING within the specific context of nerve avulsion repair, to incorporate pharmacological inhibition or rescue experiments, and to validate the gene expression results at the protein level. Overall, the present study provides a theoretical basis for STING as a novel therapeutic target in BPRA management.

## AUTHOR CONTRIBUTIONS

**Yu Peng:** Investigation. **Ying Zhang:** Investigation. **Shenhui Yang:** Investigation. **Lu He:** Conceptualization; funding acquisition; writing – original draft. **Shuangxi Chen:** Conceptualization; funding acquisition; supervision; writing – review and editing.

## FUNDING INFORMATION

This work was supported by the National Natural Science Foundation of China (grant no. 82301581), and Health Research Project of Hunan Provincial Health Commission (grant no. W20243271), grants from Natural Science Foundation of Hunan Province (grant no. 2024JJ6401).

## CONFLICT OF INTEREST STATEMENT

The authors declare no conflicts of interest.

## ETHICS STATEMENT

All animal experiments received ethical approval from The Laboratory Animal Ethics Committee of The First Affiliated Hospital of University of South China (permit no. USC2024XS286).
